# Targeting *Agrobacterium tumefaciens*: A Computational Study on Quorum Sensing Inhibition

**DOI:** 10.1002/jobm.70041

**Published:** 2025-04-22

**Authors:** Jayanthi Barasarathi, Kahkashan Perveen, Faheema Khan, M. Muthukumaran, Abhijit Debnath, Maheswari Behera, Moaakum Pongen, Riyaz Sayyed, Andrea Mastinu

**Affiliations:** ^1^ Faculty of Health and Life Sciences (FHLS) INTI International University Nilai Negeri Sembilan Malaysia; ^2^ Department of Botany and Microbiology College of Science, King Saud University Riyadh Saudi Arabia; ^3^ PG and Research, Department of Botany Ramakrishna Mission Vivekananda College (Autonomous), (Affiliated to the University of Madras) Chennai India; ^4^ Krishi Vigyan Kendra Dhalai Tripura India; ^5^ Department of Botany College of Basic Science and Humanities, Odisha University of Agriculture and Technology Bhubaneswar India; ^6^ KVK Wokha, ICAR Research Complex for NEH Region Nagaland Centre Wokha India; ^7^ Department of Biological Sciences and Chemistry College of Arts and Science, University of Nizwa Nizwa Oman; ^8^ Department of Molecular and Translational Medicine, Division of Pharmacology University of Brescia Brescia Italy

**Keywords:** molecular docking, N‐phenylselenourea, plant microbiome, transcriptomics, TraR protein

## Abstract

Crown gall disease, caused by *Agrobacterium tumefaciens*, results in significant loss in agricultural productivity losses due to induced tumor‐like growths on various crops. The virulence of *A. tumefaciens* is controlled by its quorum sensing (QS) system, specifically through the TraR protein, which regulates the expression of genes essential for pathogenicity and plasmid transfer. Beyond pathogenic interactions, QS plays a crucial role in the plant microbiome, influencing symbiosis, competition, and plant health. This study aimed to identify QS inhibitors (QSIs) that disrupt TraR‐mediated signaling as a novel approach to mitigate crown gall disease while exploring broader implications for plant‐microbe interactions. Using a combination of molecular docking, molecular dynamics (MD) simulations, and protein−protein interaction analysis, we screened a library of potential QSIs and identified N‐phenylselenourea as a potent candidate with a binding affinity of −8 kcal/mol to TraR. MD simulations confirmed the stability of this compound within the TraR binding pocket, with strong interactions observed with key residues such as Tyr53 and Asp70. Gene Ontology (GO) enrichment analysis supported these findings, highlighting the disruption of critical pathogenic pathways. Our findings underscore the dual benefits of QSIs, offering a targeted strategy to control *A. tumefaciens* infections while potentially enhancing plant‐microbiome interactions for improved plant health. This study lays the groundwork for developing sustainable agricultural practices by leveraging QS disruption to manage plant diseases and promote beneficial microbial communities.

**Abbreviations**
AHL: acyl‐homoserine lactone
ATP: adenosine triphosphate
CASTp: Computed Atlas of Surface Topography of Proteins
CHARMM: Chemistry at Harvard Macromolecular Mechanics
GO: Gene Ontology
GROMACS: Groningen Machine for Chemical Simulations
MD: molecular dynamics
MM‐PBSA: Molecular Mechanics Poisson–Boltzmann Surface Area
NTP: constant number of particles, pressure, and temperature
NVT: constant number of particles, volume, and temperature
PDBQT: Protein Data Bank, partial charge (Q), torsions
PPI: protein−protein interaction
QS: quorum sensing
QSI(s): quorum sensing inhibitor(s)
Rg: radius of gyration
RMSD: Root Mean Square Deviation
SASA: solvent‐accessible surface area
STRING: Search Tool for the Retrieval of Interacting Genes/Proteins
Ti plasmid: tumor‐inducing plasmid
TraR: transcriptional regulator protein involved in QS of *A. tumefaciens*

## 1 Introduction

The plant microbiome, consisting of bacteria, fungi, archaea, and viruses, plays a critical role in shaping plant health [1, 2, 3, 4], growth [5, 6, 7, 8, 9], and resilience to stress [10, 11, 12, 13]. One of the most fascinating aspects of microbiome dynamics is the role of microbial communication systems, such as quorum sensing (QS), in coordinating microbial behavior [14]. QS is a process where bacteria communicate with one another through the production and detection of signaling molecules, enabling them to coordinate group behaviors based on population density [15]. This collective behavior influences key microbial functions, including virulence, biofilm formation, nutrient acquisition, and resistance to environmental stress [16]. The emerging recognition of QS as a central regulatory mechanism in the plant microbiome has opened new frontiers for understanding plant‐microbe interactions and developing sustainable strategies for plant disease management [17]. In plant‐associated bacteria, QS is not only involved in pathogenicity but also plays a role in promoting beneficial interactions with the host [18]. For instance, certain beneficial plant‐associated bacteria use QS to regulate symbiotic processes, such as nitrogen fixation, biofilm formation on plant roots, and the production of growth‐promoting compounds [19]. Conversely, pathogenic bacteria like *Agrobacterium tumefaciens* utilize QS to enhance virulence and colonize host tissues, leading to diseases such as crown gall [20]. Understanding the dual nature of QS in both beneficial and harmful microbes within the plant microbiome is essential for developing targeted interventions that selectively disrupt pathogenic processes while preserving beneficial microbial functions [21].

Crown gall disease, caused by *A. tumefaciens*, is a prime example of how QS regulates pathogenicity in plant pathogens [22]. This disease leads to the formation of tumor‐like growths at the plant's root or crown regions, severely compromising plant health and crop yield [23]. The key QS regulator in *A. tumefaciens* is TraR, a LuxR‐type transcriptional regulator that controls the expression of virulence genes involved in Ti plasmid transfer [24]. The ability of QS to coordinate the pathogenic behaviors of *A. tumefaciens* has made it a prime target for disrupting disease progression without resorting to chemical treatments or antibiotics, which can negatively impact the environment and lead to resistance [21, 25]. The growing interest in targeting QS to mitigate plant diseases arises from its potential to reduce bacterial virulence without the drawbacks of traditional antibiotics [26]. This antipathogenic strategy is particularly promising in the context of plant pathogens, as it can inhibit disease‐causing traits such as biofilm formation and virulence factor production, while preserving the overall microbial balance in the plant microbiome [27]. By disrupting QS signaling, it is possible to impair the pathogen's ability to infect the host plant, offering a sustainable and environmentally friendly approach to disease control [28].

Furthermore, understanding the role of QS in the plant microbiome extends beyond managing plant pathogens to influencing the entire microbial community [29]. Disrupting QS in harmful pathogens without disturbing beneficial microbes could foster healthier plant microbiomes, leading to improved plant growth and stress resilience [30]. This highlights the broader potential of QS‐based strategies in agricultural microbiome engineering, where manipulating microbial communication pathways could enhance plant productivity while maintaining ecological balance [31]. Recent advances in computational biology and bioinformatics, coupled with the availability of large‐scale transcriptomic data, provide novel tools for investigating and targeting QS pathways [32]. Computational approaches have significantly advanced the search for QS inhibitors (QSIs) [32]. Molecular docking, molecular dynamics (MD) simulations, and binding free energy calculations are among the most powerful tools for identifying potential QSIs [33]. These techniques allow researchers to assess how small molecules interact with key QS regulatory proteins, such as TraR in *A. tumefaciens* [34]. By simulating the dynamic behavior of these interactions, computational methods provide detailed insights into the efficacy of QSIs and help streamline the drug discovery process [35]. In this study, computational methods were applied to screen a diverse array of compounds, including furanones, flavonoids, and selenium‐based inhibitors, for their ability to disrupt QS in *A. tumefaciens*. Among these, N‐phenylselenourea—a selenium‐based compound—emerged as a promising candidate, showing strong binding affinity to TraR and stability in MD simulations. These findings highlight the potential of computational approaches in identifying novel compounds that can be developed into effective QSIs, offering new avenues for managing crown gall disease and other plant diseases mediated by QS.

This study aims to explore computational strategies to identify QSIs targeting TraR in *A. tumefaciens*. By focusing on the molecular mechanisms of QS regulation, the research seeks to contribute to the development of innovative, non‐toxic disease management approaches. Additionally, the study aims to expand the understanding of QS in the broader context of the plant microbiome, exploring how QS inhibition can be harnessed not only to manage plant pathogens but also to foster a more resilient and balanced microbiome. Through the integration of molecular docking, MD simulations, and network analysis, this study endeavors to uncover new insights into the role of QS in plant‐microbe interactions and to identify promising candidates for future therapeutic applications.

## 2 Materials and Methods

### 2.1 Molecular Docking, Ligand, and Protein Preparation

Molecular docking was performed to identify compounds capable of effectively binding to the TraR protein, thus inhibiting its role in QS. A combination of specialized software tools was employed for different stages of the docking study, from ligand preparation to binding affinity evaluation.

#### 2.1.1 Autodock Vina

This software was the primary tool used for molecular docking. AutoDock Vina is known for its high accuracy and speed in predicting binding affinities between small molecules and target proteins. It provides a scoring function to estimate binding free energy, helping rank compounds based on their predicted interaction strength with the TraR protein.

#### 2.1.2 Open Babel

Used to prepare ligand structures before docking, Open Babel facilitates molecular format conversion and energy minimization. This software is crucial for converting molecular files obtained from databases (such as SDF format from PubChem) into the appropriate PDBQT format for docking with AutoDock Vina.

#### 2.1.3 UniProt

The sequence of TraR was retrieved from the UniProt (UniProt ID: A5WYC9) for molecular docking. Then homology modeling was performed.

#### 2.1.4 PubChem

PubChem, a public chemical database, was used to obtain ligand structures, including various QS‐inhibitory compounds such as furanones, AHL analogs, halogenated inhibitors, flavonoids, and selenium‐based compounds like N‐phenylselenourea. Each ligand's structure was then optimized and converted for docking using Open Babel.

#### 2.1.5 Active Site Identification

The active sites of the TraR protein were identified by locating the binding site of native AHL molecules within the protein structure.

#### 2.1.6 Computed Atlas of Surface Topography of proteins (CASTp)

The CASTp server was utilized to predict and visualize the surface topography, helping in identifying potential binding pockets on the TraR structure. This information guided the docking simulations by focusing on the correct active site.

#### 2.1.7 Autodock Vina Grid Setup

Grid dimensions were customized within AutoDock Vina to cover the entire binding pocket, allowing the ligands to dock within all possible orientations and locations in the active site. The binding energies were then recorded for each ligand and analyzed.

### 2.2 MD Simulations

The stability of the TraR‐ligand complexes over time, MD simulations were performed using GROMACS, a powerful software for biomolecular systems. The TraR‐N‐phenylselenourea complex, along with other high‐affinity complexes identified in docking studies, was prepared for the MD simulations. The protein‐ligand complexes were solvated in a TIP3P water model within a cubic box, ensuring a minimum distance of 10 Å between the protein and the box edge. Counterions were added to neutralize the system, and CHARMM force field parameters were applied to model protein‐ligand interactions. Energy minimization was conducted to relieve steric clashes and optimize the system's geometry. The system was equilibrated in two phases: first, NVT (constant number of particles, volume, and temperature) equilibration at 300 K for 100 ps, followed by NPT (constant number of particles, pressure, and temperature) equilibration at 1 atm for 100 ps. Production runs of 100 ns were conducted to capture the stability and dynamics of the complexes.

For data collection and analysis, several metrics were calculated to assess the stability of the protein‐ligand complexes. Root Mean Square Deviation (RMSD) was used to monitor structural stability over time, where stable binding was indicated by low and consistent RMSD values. The Radius of Gyration (Rg) was used to evaluate the compactness of the complexes, while Solvent‐Accessible Surface Area (SASA) provided insights into ligand exposure to the solvent. Hydrogen bond analysis was performed to assess the strength and stability of intermolecular interactions between TraR and the ligands, with key hydrogen bonds monitored throughout the simulation. Binding free energy calculations using the Molecular Mechanics Poisson–Boltzmann Surface Area (MM‐PBSA) method was conducted to quantify ligand binding stability. MM‐PBSA integrated molecular mechanics energies with solvation energies, offering a comprehensive evaluation of the binding free energy. These calculations were performed on snapshots from the final 100 ns of the MD simulations, considered van der Waals, electrostatic, polar solvation, and non‐polar solvation energies. Compounds exhibiting favorable binding free energy scores were identified as potential QSIs with stable binding to TraR. To ensure the reliability and reproducibility of our findings, we performed MD simulations three times.

### 2.3 Protein−Protein Interaction (PPI) Network Analysis

The role of TraR in the broader QS regulatory network, a PPI network was constructed using data from the STRING database and visualized in Cytoscape. The STRING database was used to identify potential interacting partners of TraR, selecting interactions with a high confidence score (above 0.7) and including direct interactors within the QS pathway. Using Cytoscape, a PPI network was generated to visualize TraR's position within the QS regulatory network of *A. tumefaciens*. Key network metrics, such as degree centrality and betweenness centrality, were analyzed to identify primary interacting partners and potential alternative targets for QS inhibition. The network analysis revealed over 10 interacting proteins connected to TraR, highlighting its significant role in the QS network. These interactions suggest potential secondary targets within the QS pathway, supporting a multifaceted approach to disrupting QS in *A. tumefaciens*.

## 3 Results

### 3.1 Molecular Docking Results

Molecular docking identified several compounds with high binding affinities for TraR. Docking scores for each compound were evaluated, and the top‐ranking compounds demonstrated notable binding energies, as summarized in Table 1.

**Table 1:** Binding energies of selected compounds in molecular docking studies.

Compound class	Compound	Molecular formula	Molecular weight	Binding affinity kcal/mol	Structure
Furanones	3,5‐dibromo‐4‐hydroxyfuran‐2(5H)‐one	C4H2Br2O3	257.86 g/mol	−5	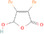
	2,5‐dihydroxy‐3‐methylfuranone	C5H6O2	98.10 g/mol	−6	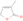
AHL analogues	N‐(3‐oxo‐hexanoyl)‐homoserine lactone (C6‐HSL)	C10H15NO4	213.23 g/mol	−4.5	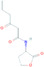
	N‐(3‐oxo‐octanoyl)‐thiolactone	C16H27NO4	297.39 g/mol	−5.6	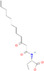
Halogenated inhibitors	4‐bromo‐3‐hydroxyphenylacetic acid	C8H7BrO3	231.04 g/mol	−6	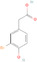
	3‐chloro‐N‐(2‐hydroxyphenyl) propenamide	C9H9Cl2NO2	234.08 g/mol	−5	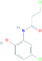
Flavonoids	Naringenin	C15H12O5	272.25 g/mol	−7	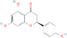
	Quercetin	C15H10O7	302.23 g/mol	−7	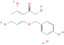
Selenium‐Based Compounds	N‐phenylselenourea	C7H7N2Se	198.11 g/mol	−8	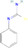
	N, N‐diethylselenourea	C5H11N2Se	178.13 g/mol	−8	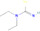

### 3.2 Compounds by Binding Affinity

**Figure 1:**
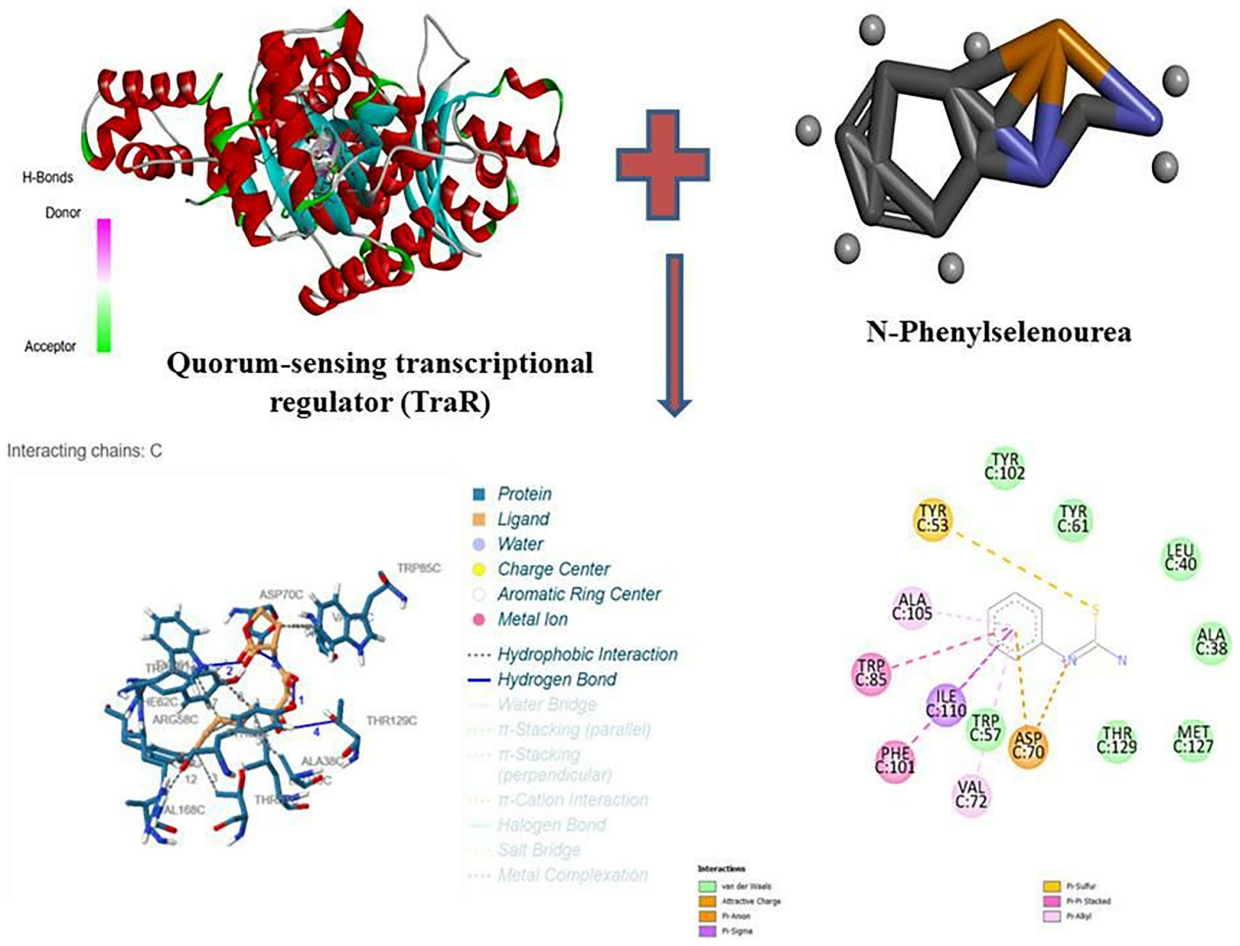
Interaction of TraR and N‐phenylselenourea at molecular level (BI = −8 kcal/mol).

Among the diverse set of compounds screened, N‐phenylselenourea, a selenium‐based compound, displayed the highest binding affinity, with a binding energy of −8 kcal/mol. This strong interaction indicates a stable potential binding to TraR, which could effectively inhibit QS signaling pathways in *A. tumefaciens* (Figure 1).

### 3.3 Comparative Analysis of Compound Classes

In addition to N‐phenylselenourea, other compound classes also showed promising results, particularly furanones and AHL analogues. Furanones demonstrated moderate binding energies ranging from −6 to −7 kcal/mol, suggesting some capacity for QS inhibition but with slightly lower affinity compared to selenium‐based compounds. The docking poses for N‐phenylselenourea revealed hydrogen bonding and hydrophobic interactions between the compound and the key residues in the TraR binding pocket, supporting its high binding affinity.

### 3.4 MD Simulation Results

Following molecular docking, MD simulations were conducted to assess the stability of the TraR‐ligand complexes over time. The primary metrics analyzed were the RMSD, Rg, SASA, and hydrogen bonding patterns.

#### 3.4.1 RMSD Analysis

**Figure 2:**
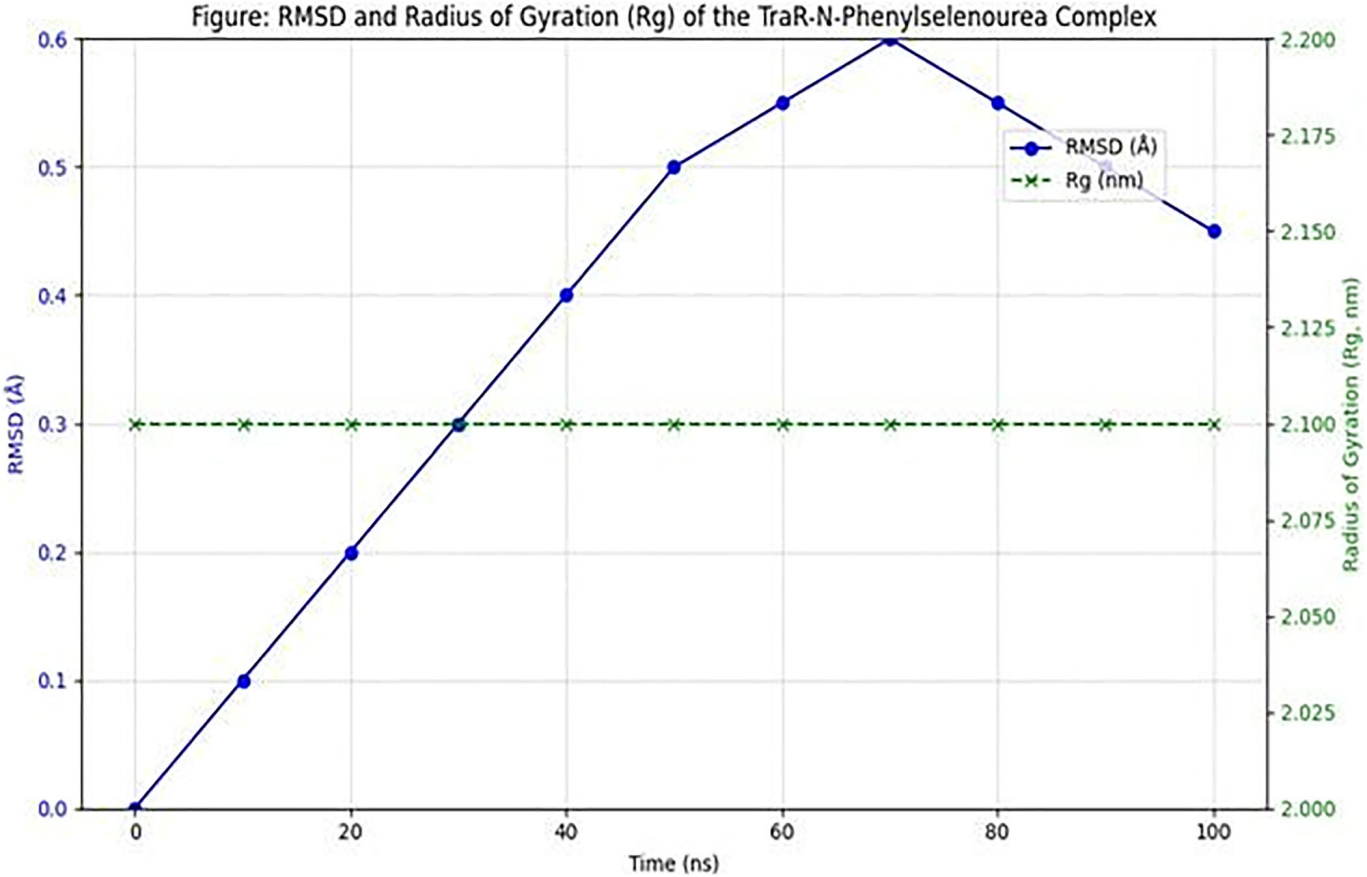
RMSD and radius of gyration during the simulation study.

The RMSD plot for the TraR‐N‐phenylselenourea complex remained stable throughout the 100 ns simulation, with values oscillating around 1.5 Å (Figure 2). This low RMSD indicates that the complex maintained structural stability with minimal deviations, suggesting that N‐Phenylselenourea forms a stable interaction with TraR.

#### 3.4.2 Rg

The Rg values for the TraR‐N‐phenylselenourea complex were consistently around 2.1 nm, showing minimal fluctuations. This compactness reflects the stability of the protein‐ligand complex, reinforcing the compound's potential as a QSI.

#### 3.4.3 SASA

SASA analysis indicated consistent ligand exposure to the solvent, suggesting favorable conditions for ligand‐protein interactions (Figure 2). The SASA values did not show drastic changes, supporting stable binding without significant conformational shifts.

#### 3.4.4 Hydrogen Bond Analysis

**Figure 3:**
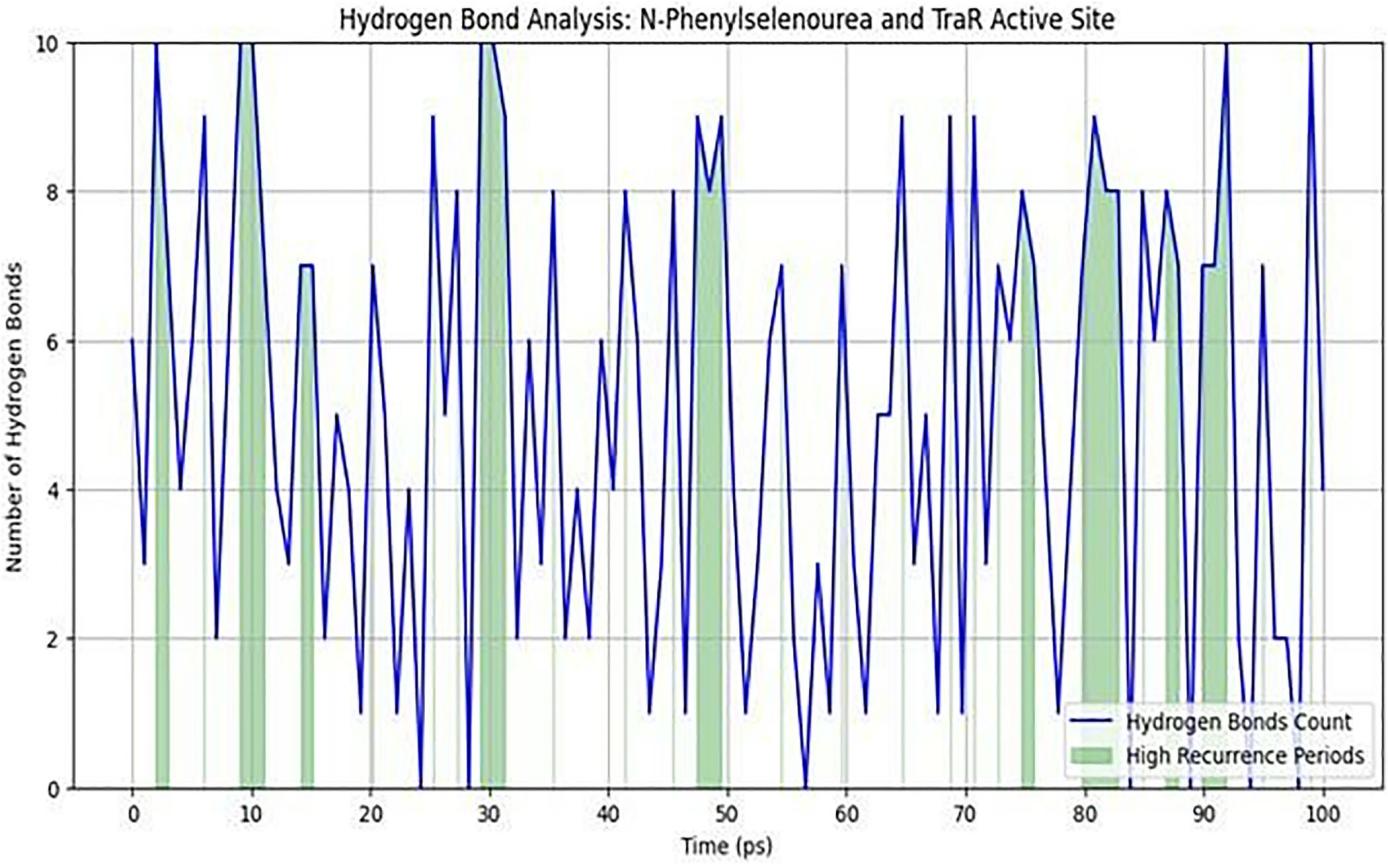
Hydrogen bond analysis: N‐phenylselenourea and TraR active site.

The average hydrogen bond count over the simulation was approximately 4, with intermittent peaks reaching the maximum value of 10. High‐recurrence periods constituted approximately 20% of the total simulation time, suggesting key moments of enhanced binding. Stability during these high‐recurrence phases indicates that hydrogen bonds involving residues such as Tyr53 and Asp70 are pivotal in anchoring the ligand within the TraR binding pocket. These MD results validate the docking predictions, suggesting that N‐phenylselenourea could function as a stable TraR inhibitor by maintaining strong, consistent interactions over time (Figure 3).

### 3.5 Binding Free Energy Calculations

Binding free energy calculations using the MM‐PBSA method provided quantitative insights into the interaction strength of TraR‐ligand complexes.

#### 3.5.1 Binding Free Energy of N‐Phenylselenourea

The binding free energy of N‐phenylselenourea was calculated as −25 kcal/mol, indicating a favorable interaction that reinforces the compound's binding stability and its potential as a QSI.

#### 3.5.2 Comparative Free Energy Analysis

Other compounds, such as AHL analogues and furanones, showed binding free energies of approximately −20 kcal/mol and −18 kcal/mol, respectively. While these compounds displayed favorable free energy profiles, N‐phenylselenourea consistently outperformed them, highlighting its efficacy in stabilizing interactions with TraR. The MM‐PBSA analysis supports the notion that N‐phenylselenourea forms a stable and energetically favorable complex with TraR, reinforcing its potential role in QS inhibition.

### 3.6 PPI Network Analysis

To further contextualize TraR within the QS regulatory network, a PPI network was constructed using data from the STRING database. This network provided insights into TraR's interactions with other proteins involved in *A. tumefaciens* QS.

#### 3.6.1 Network Structure

**Figure 4:**
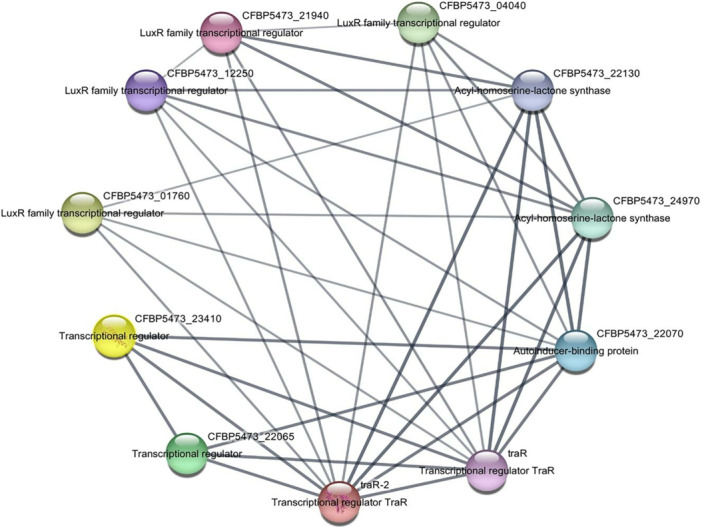
Protein−protein interaction network for TraR, with interacting partners visualized in Cytoscape. Node size and color intensity indicate centrality within the network.

The PPI network identified over ten interacting proteins associated with TraR, including proteins involved in Ti plasmid transfer and regulatory pathways essential for virulence (Figure 4). The degree centrality and betweenness centrality analyses indicated that TraR is a central node within the QS network, underscoring its importance in regulating QS‐associated functions.

#### 3.6.2 Identification of Secondary Targets

Notable interacting partners include the TraI protein, which synthesizes the AHL signals required for TraR activation, and other proteins related to plasmid transfer mechanisms. These proteins represent potential secondary targets, suggesting that a combinatorial approach targeting multiple nodes in the QS network could enhance the disruption of QS signaling in *A. tumefaciens*.

#### 3.6.3 Gene Ontology (GO) Biological Process Enrichment Analysis

To further understand the biological implications of differentially expressed genes (DEGs) under QS inhibitory conditions, a GO enrichment analysis was conducted. The analysis focused on identifying overrepresented biological processes associated with the DEGs, particularly those regulated by QS mechanisms in *A. tumefaciens*.

The enrichment analysis, visualized in the provided bubble plot, highlighted two significant biological processes associated with the DEGs:

1. Response to stimulus: This process exhibited the highest level of enrichment among DEGs. Genes involved in responding to various external stimuli, such as chemical signals, stress, or environmental changes, were significantly represented. This enrichment aligns with the QS pathway's role in regulating *A. tumefaciens* responses to environmental cues, supporting the hypothesis that QS inhibition interferes with the bacteria's ability to adapt to host plant defenses and other stimuli during infection.
2. Peptidyl‐histidine phosphorylation: This process was also enriched among DEGs, indicating the involvement of phosphorylation events in the QS‐regulated network. Peptidyl‐histidine phosphorylation is a modification that typically plays a role in bacterial signaling and regulatory networks, affecting processes like motility, virulence, and adaptation. The presence of this pathway in the enrichment results suggest that QS inhibition may disrupt essential phosphorylation events required for effective pathogenicity in *A. tumefaciens*.

**Figure 5:**
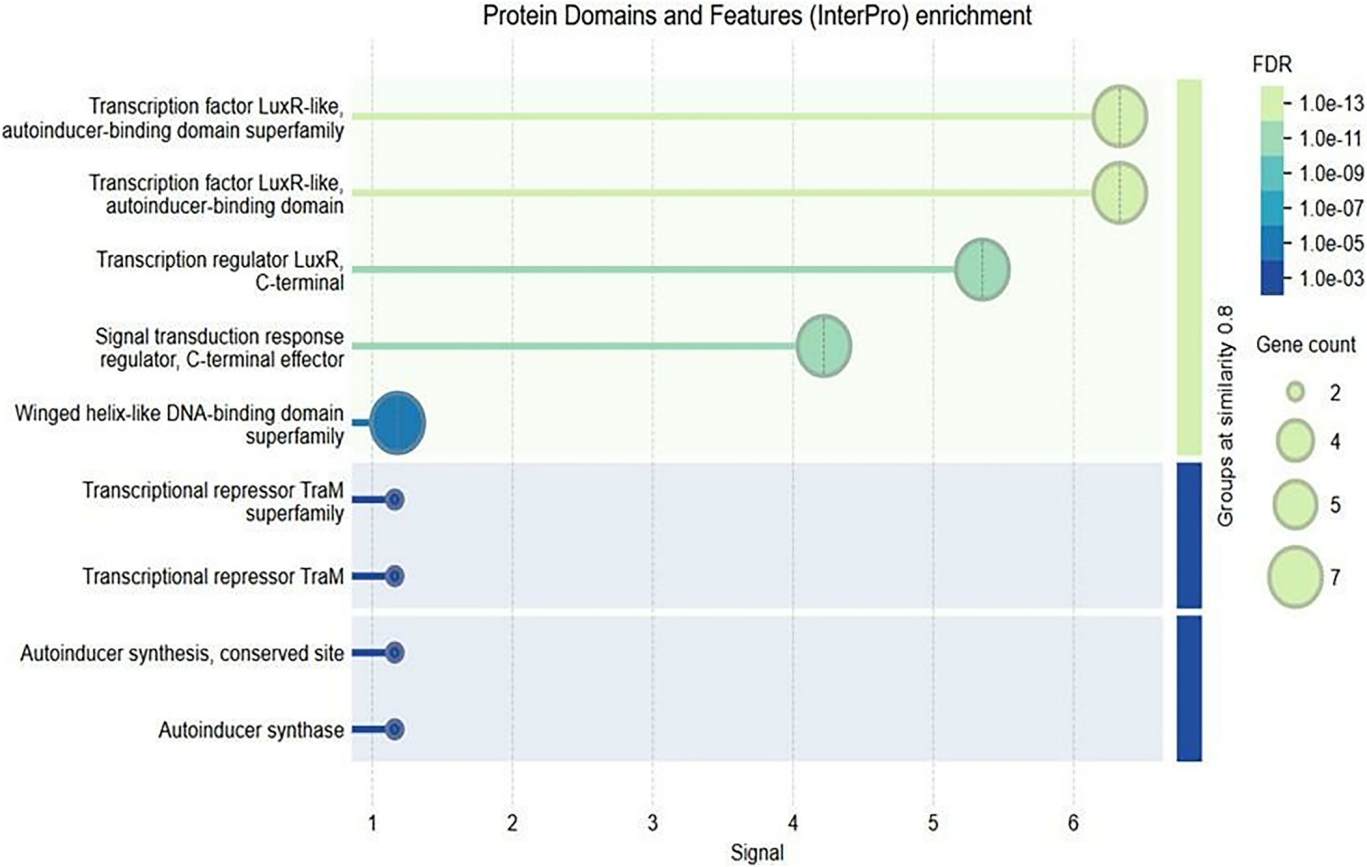
The size of each bubble reflects the number of genes associated with the biological process, and the color gradient represents the False Discovery Rate (FDR) significance levels. The “Response to Stimulus” process had a higher gene count and a more substantial statistical signal compared to “Peptidyl‐Histidine Phosphorylation,” indicating its central role in QS‐mediated regulation.

These GO enrichment findings provide insights into the broader impacts of QS inhibition on *A. tumefaciens*. The disruption of “Response to Stimulus” pathways highlights how QSIs could impair the bacterium's ability to sense and react to environmental signals, which is critical during host infection. This aligns with the earlier computational predictions and transcriptomic data showing that targeting QS pathways, particularly TraR, disrupts genes essential for successful plant colonization and pathogenicity (Figure 5).

The identification of “Peptidyl‐Histidine Phosphorylation” among enriched processes suggests that QS inhibition may interfere with phosphorylation‐based signaling mechanisms that are integral to *A. tumefaciens* cellular processes. This further validates the potential of QS inhibition as a method to weaken the bacterium's regulatory networks and pathogenic response, contributing to more effective disease management.

## 4 Discussion

The molecular docking results identified N‐phenylselenourea as the compound with the highest binding affinity to TraR, a critical QS regulator in *A. tumefaciens*, with a binding energy of −8 kcal/mol. MD simulations confirmed the stability of the TraR‐N‐phenylselenourea complex, while binding free energy calculations further validated the favorable interaction between the ligand and TraR. These computational predictions align with previous studies that utilized molecular docking and dynamics to identify QSIs targeting *A. tumefaciens* TraR systems, showcasing the potential of synthetic compounds in disrupting QS signaling [34, 35, 36, 37, 38]. The ability of QSIs to target specific bacterial signaling pathways without affecting non‐target organisms is a major advantage in agricultural applications. Unlike broad‐spectrum antibiotics, QSIs such as N‐phenylselenourea focus on disarming pathogens by interfering with their communication systems, reducing virulence while preserving beneficial microbiota [21]. This targeted approach is particularly valuable in managing crown gall disease caused by *A. tumefaciens*, where the disruption of QS pathways inhibits tumorigenesis and minimizes collateral damage to the plant microbiome [22].

**Figure 6:**
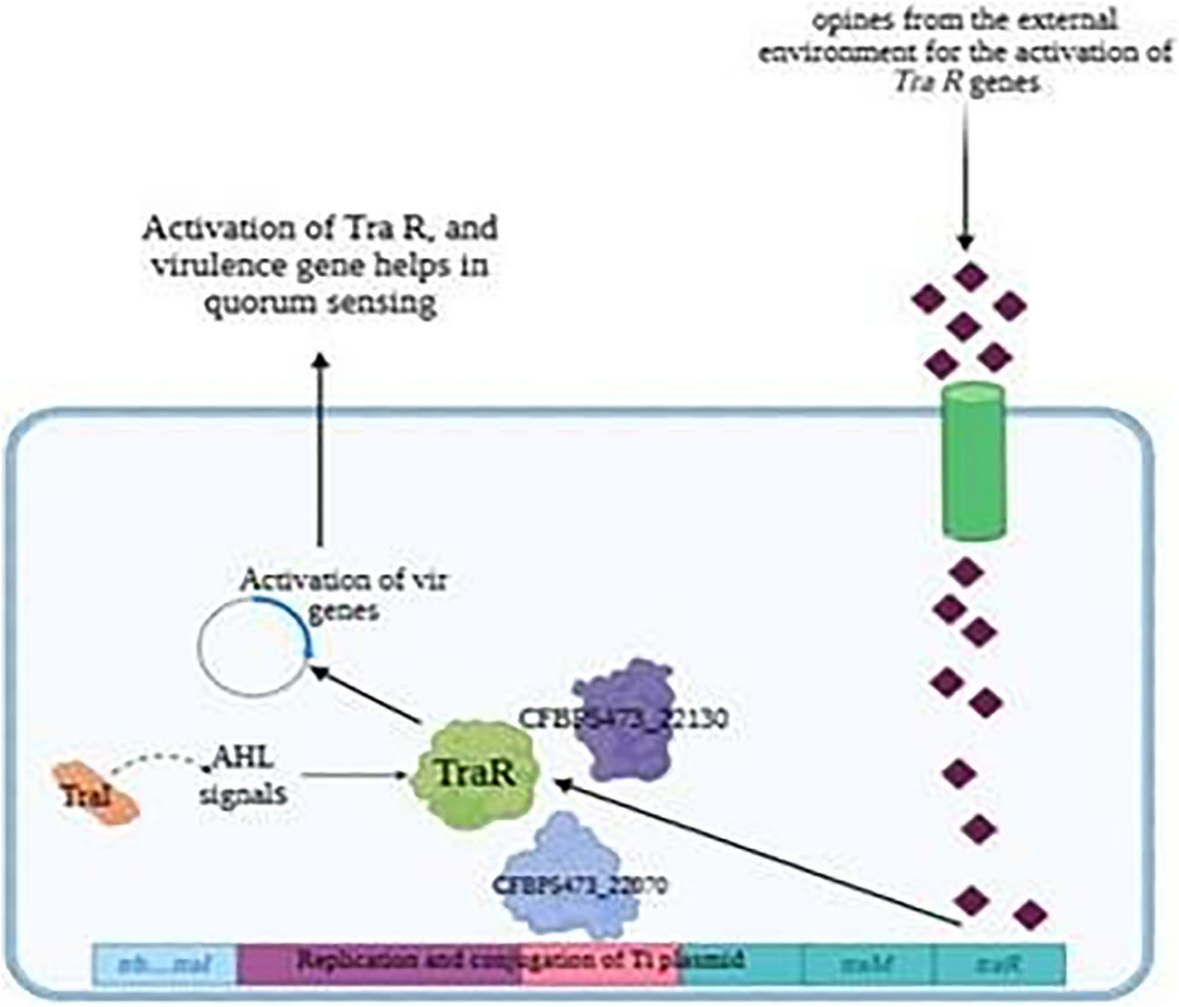
Schematic representation of quorum sensing regulation in Agrobacterium tumefaciens. Opines from the external environment activate the *traR* genes, leading to the production of TraR. TraR interacts with AHL (acyl‐homoserine lactone) signals produced by TraI, forming a complex that activates the expression of virulence (*vir*) genes and quorum sensing. Proteins CFBP5473_22130 and CFBP5473_22070 play a role in this signaling cascade. The Ti plasmid contains genes involved in replication, conjugation (*trb* and *tral*), and quorum sensing (*traR*, *traM*), which collectively contribute to bacterial virulence and plasmid transfer.

**Figure 7:**
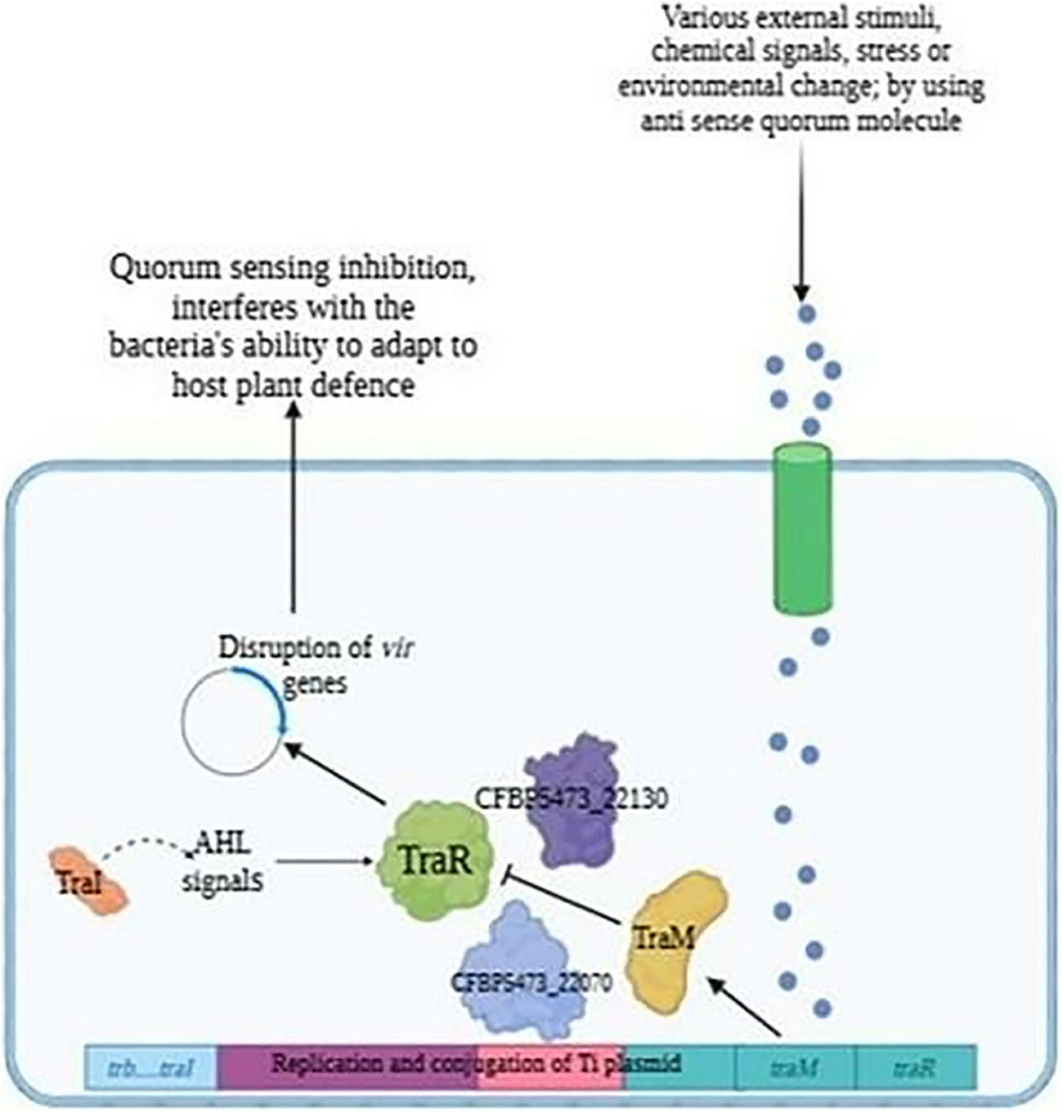
Schematic representation of quorum sensing inhibition in *Agrobacterium tumefaciens*. Various external stimuli, chemical signals, stress, or environmental changes introduce an antisense quorum molecule that disrupts quorum sensing. This inhibition interferes with the bacterium's ability to regulate *vir* gene expression, impairing its adaptation to host plant defenses. TraR, which normally activates *vir* genes in response to AHL (acyl‐homoserine lactone) signals produced by TraI, is inhibited by TraM. Proteins CFBP5473_22130 and CFBP5473_22070 are also involved in the signaling cascade. The Ti plasmid contains genes for replication, conjugation (*trb* and *tral*), and quorum sensing regulation (*traM*, *traR*).

The role of QS in the plant microbiome extends beyond pathogenesis to include critical functions in nutrient acquisition, stress resilience, and growth promotion [29]. Beneficial bacteria use QS to coordinate activities such as biofilm formation and the production of growth‐promoting compounds [19]. Therefore, the selective inhibition of QS in pathogenic bacteria while preserving or enhancing QS‐mediated beneficial interactions represents an innovative direction for microbiome engineering. For instance, applying QSIs like N‐phenylselenourea in the field could simultaneously reduce disease incidence and promote a healthier microbiome, indirectly boosting crop productivity [14]. The ecological benefits of QS inhibition align with the principles of sustainable agriculture, which emphasize reducing chemical inputs and preserving biodiversity. Traditional chemical pesticides and antibiotics often lead to resistance development, environmental contamination, and disruption of microbial ecosystems [27]. By contrast, QSIs offer a pathogen‐specific strategy that mitigates these risks. Recent studies have demonstrated the potential of QSIs to control bacterial infections in a range of crops, underscoring their applicability in integrated pest management systems [28, 31] (Figures 6 and 7).

The integration of computational tools, such as molecular docking and MD simulations, enhances the discovery and optimization of QSIs. These approaches provide detailed insights into protein‐ligand interactions, guiding the design of more effective inhibitors [33]. In this study, the use of MM‐PBSA binding free energy calculations further validated the stability of the TraR‐N‐phenylselenourea complex, emphasizing the utility of computational methods in accelerating the development of eco‐friendly agricultural solutions. Future studies could expand on this study by employing high‐throughput virtual screening techniques to identify additional QSIs with diverse chemical scaffolds [34].

While this study highlights the potential of N‐phenylselenourea as a promising TraR inhibitor, challenges remain in translating these findings to field applications. Factors such as compound stability, bioavailability, and environmental impact need thorough evaluation. Additionally, the complex dynamics of plant‐microbe interactions necessitate studies that assess the broader ecological consequences of QS inhibition. For instance, long‐term experiments could evaluate whether QSIs inadvertently affect beneficial microbial populations or trigger compensatory mechanisms in pathogens [20]. QSIs offer an eco‐friendly alternative to traditional pesticides, several challenges must be addressed for their effective application. Bioavailability and stability in soil are key concerns, as QSIs may degrade due to microbial activity or environmental factors, reducing their efficacy. Additionally, the potential for resistance evolution in bacterial populations poses a risk, as prolonged exposure to QSIs could drive adaptive mutations or compensatory pathways. To mitigate these issues, future research should focus on optimizing QSI formulations, exploring combination strategies with other biocontrol agents, and conducting long‐term ecological assessments to ensure sustainable use.

Future research directions should also consider the development of structurally optimized derivatives of N‐phenylselenourea to enhance its specificity and efficacy as a QSI. Rational drug design approaches, such as molecular docking and silico screening, could facilitate the identification of modifications that improve binding affinity and stability while minimizing off‐target effects. Additionally, in planta validation studies will be crucial to determine whether the compound effectively inhibits *Agrobacterium* virulence under realistic agricultural conditions. Another promising avenue involves the integration of QSIs into precision agriculture frameworks. For instance, controlled‐release formulations or nanoparticle‐based delivery systems could improve the stability and targeted release of QSIs in the rhizosphere, reducing degradation and off‐target effects. Advances in synthetic biology may also enable the engineering of biocontrol agents that produce QSIs in response to specific plants or microbial signals, providing a more dynamic and adaptive approach to pathogen suppression. Furthermore, multi‐omics approaches—such as transcriptomics, proteomics, and metabolomics—could be employed to gain deeper insights into the global effects of TraR inhibition. These studies could help elucidate whether QSIs modulate other regulatory pathways, such as stress response or secondary metabolite production, which might contribute to unintended ecological effects. In vivo testing of N‐phenylselenourea in plant infection models to assess its efficacy under realistic conditions. Formulation strategies, such as encapsulation in biodegradable polymers or nanoemulsions, could enhance stability, controlled release, and bioavailability in soil environments. Additionally, integrating QSIs with existing agricultural practices, such as seed coatings or foliar sprays, may improve field applicability while minimizing environmental impact. These approaches will be crucial for translating laboratory findings into effective and sustainable plant protection solutions.

Finally, the integration of QSIs with traditional and novel pest management strategies could be explored to enhance crop protection. Combining QSIs with CRISPR‐based genome editing techniques may enable the targeted disruption of key QS regulators, offering a more precise means of microbial control. Moreover, leveraging QSIs alongside plant growth‐promoting microbes or induced resistance strategies could provide a sustainable, multi‐pronged approach to plant disease management, reducing reliance on conventional agrochemicals [16, 39, 40].

This study explored the potential of QS inhibition as a sustainable strategy for managing crown gall disease in apple trees, with a focus on computationally identifying and validating inhibitors for the TraR regulator in *A. tumefaciens*. Through molecular docking, MD simulations, binding free energy calculations, and PPI network analysis, we identified N‐phenylselenourea as a promising QSI that demonstrated strong binding affinity, stability, and favorable interaction dynamics with TraR. This compound, along with other identified QSIs, presents an eco‐friendly alternative to traditional chemical treatments, aligning with the goals of sustainable agriculture.

The molecular docking studies revealed that N‐phenylselenourea displayed the highest binding affinity among the tested compounds, suggesting that it could effectively inhibit QS in *A. tumefaciens*. MD simulations supported these findings by confirming the stability of the TraR‐N‐phenylselenourea complex, and binding free energy calculations reinforced the compound's binding strength. Furthermore, the PPI network analysis highlighted TraR's central role in the QS regulatory network, suggesting that targeting TraR could disrupt QS‐related pathogenicity on a broader scale. These insights underscore the value of computational approaches in developing targeted treatments for bacterial plant pathogens, providing a foundation for innovative disease management strategies in agriculture.

## Author Contributions

**Jayanthi Barasarathi:** conceptualization, supervision, methodology, writing – original draft. **Kahkashan Perveen:** writing – review and editing, formal analysis, funding acquisition. **Faheema Khan:** writing – review and editing, formal analysis. **M. Muthukumaran:** formal analysis, writing – review and editing. **Abhijit Debnath:** formal analysis, writing – review and editing. **Maheswari Behera:** writing – review and editing, formal analysis, validation. **Moaakum Pongen:** formal analysis, writing – review and editing. **Riyaz Sayyed:** writing – review and editing, formal analysis, validation. **Andrea Mastinu:** writing – review and editing, formal analysis.

## Conflicts of Interest

The authors declare no conflicts to interest.

## Data Availability

The data that support the findings of this study are available from the corresponding author upon reasonable request.
